# The Effect of Mineral Trioxide Aggregate on the Periapical Tissues after Unintentional Extrusion beyond the Apical Foramen

**DOI:** 10.1155/2016/3590680

**Published:** 2016-10-20

**Authors:** Pradnya S. Nagmode, Archana B. Satpute, Ankit V. Patel, Pushpak L. Ladhe

**Affiliations:** Department of Conservative Dentistry and Endodontics, SMBT Dental College & Hospital, Sangamner, India

## Abstract

*Introduction*. Single-step apexification procedures using mineral trioxide aggregate (MTA) have been reported as favorable treatment options for teeth with an open apex, posing greater benefits compared to the other available medicaments. However, controlled apical placement of MTA is a challenging procedure to perform using orthograde approach. This case series describes the outcome of the unintentional extrusion of MTA into periradicular tissues during apexification, in three separate cases.* Methods*. Three adult patients reported to the Department of Conservative Dentistry and Endodontics for the management of maxillary incisors with open apices. After isolation, conventional access, and cleaning and shaping procedures, one-step MTA apexification was performed. On subsequent radiographs, a considerable amount of MTA was seen to be extruded in all the three cases.* Results*. During follow-up examination the teeth were seen to be asymptomatic in all cases and radiographically demonstrated repair of the periapical lesion.* Conclusion*. The results of these cases suggest that extrusion of MTA into the periapical tissues does not cause any detrimental effect, which could be attributed to the biologic properties of MTA.

## 1. Introduction

The conventional school of thought regarding the clinical treatment of a traumatized or an infected immature vital tooth is preserving the vitality by apexogenesis or revascularization so as to allow complete root formation. However, such teeth with nonvital pulps require a hard-tissue apical barrier for the completion of the root filling [1, 2].

Earlier, intracanal medicaments were used over several appointments to treat cases with open apices, with the hope of creating a barrier over which gutta-percha could eventually be placed. The treatment could be prolonged to a year, with little evidence of an apical barrier, resulting in thinner and brittle roots which are more susceptible to fracture [3].

Mineral trioxide aggregate (MTA) has evolved as a material of choice for apexification owing to its properties of better biocompatibility, good sealability, ability to promote dental pulp, and periradicular tissue regeneration. Moreover, it can also harden in the presence of moisture [4]. Apexification with MTA can be performed as a one- or two-visit procedure, eliminating the need for extended periods of dressing with calcium hydroxide. It also allows for immediate restoration, which further reduces the potential of catastrophic, vertical, or oblique root fractures of such teeth [5].

However, MTA has been known to exhibit certain shortcomings, which include long setting time, the potential to discolour teeth, technique sensitivity, and high cost [6, 7]. Also, any variation in pH as a result of inflammatory changes in periradicular lesions may adversely affect the physical, chemical, and hydration properties of MTA [8].

The lack of normal apical constriction of the root canal in teeth with open apices complicates the procedures for apexification. The wide apical foramen requires a large volume of filling material that may extrude into the periradicular tissues. This produces undesirable tissue inflammation, neurotoxic effects, and foreign body reaction [1]. Overfillings have a deleterious effect on the prognosis of root canal treatments; the teeth obturated within 0–2 mm from the apex have a success rate of 94%, which drops down to 76% if the teeth are overfilled [9]. The response of periapical tissues to the root filling material, as a consequence of the complex and dubious interaction between the materials and the host defences, determines the prognosis for an endodontically treated tooth which has been overfilled [10].

This case series describes the outcomes of unintentional extrusion of MTA into the periradicular tissues during apexification, in three separate cases.

## 2. Case Reports

### 2.1. Case 1

**Figure 1:**
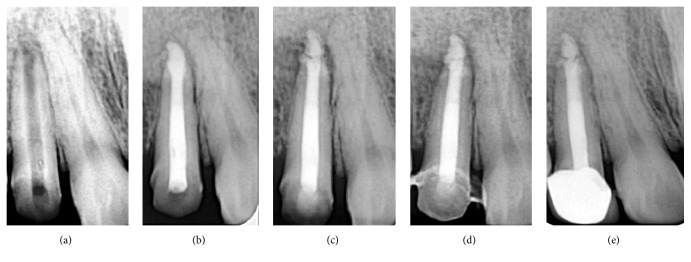
(a) Preoperative IOPA, (b) unintentional extrusion of MTA from apical foramen, (c) 1-month follow-up, (d) 3-month follow-up, and (e) 6-month follow-up.

A 29-year-old female patient reported to the Department of Conservative Dentistry and Endodontics for endodontic retreatment of the right maxillary lateral incisor. The chief complaint was pain with tooth #12. She presented with a history of root canal treatment with the same tooth 2 years back. Medical history was not contributory. On clinical examination, the tooth was tender to percussion. A localized, erythematous swelling could be appreciated on the buccal mucosa surrounding the tooth. Radiographically, tooth #12 showed periapical rarefaction measuring approximately 2 mm (width) × 4 mm (height), with a resorbed apex (Figure 1(a)). A diagnosis of chronic apical abscess was made, and endodontic retreatment was planned.

Following placement of the rubber dam, endodontic access cavity was modified and the gutta-percha was removed using xylene (RC Solve; Prime Dental Products Pvt. Ltd., Thane, India) and ProFile rotary instruments. The canal was instrumented with K-files up to size #50, irrigated with 5.25% NaOCl (Prime Dental Products Pvt. Ltd., Thane, India), and dressed with calcium hydroxide (RC Cal; Prime Dental Products Pvt. Ltd., Thane, India). The entire length of a size #40 Lentulo spiral (Maillefer, Ballaigues, Switzerland) was coated with the paste, introduced into the canal to the working length, and then rotated at 500 rpm using a handpiece mounted on a speed and torque control machine (X-smart, Dentsply, Tulsa Dental, Tulsa, OK, USA). After ten days, the tenderness had disappeared, and no soft tissue swelling, erythema, or canal exudate were seen. The temporary restoration was then removed under rubber dam isolation and local anaesthesia, and the calcium hydroxide dressing was flushed out using alternating irrigation with 5.25% NaOCl and 17% EDTA (Prime Dental Products Pvt. Ltd., Thane, India) together with gentle filing with a size of 50 K file. Since no exudate was found on the blotted paper points test, a one-step apexification procedure with MTA was performed. MTA (ProRoot MTA; Dentsply Tulsa Dental Specialties, Johnson City, TN, USA) was manipulated according to the manufacturer's instructions, carried to the apical 4 mm of the canal using an MTA carrier, and then condensed with measured endodontic pluggers under an operating dental microscope (Seiler IQ: St. Louis, MO). Radiograph taken during the procedure displayed considerable extrusion of the MTA into the periradicular tissues (Figure 1(b)). No attempt was made to remove the extruded MTA, and the access cavity was sealed with sterile cotton pellets and temporary restorative material (Cavit, 3M/ESPE, Seefeld, Germany). The patient was kept under observation.

At the following appointment the cotton pellet was removed; the setting of MTA and other clinical signs and symptoms were verified. The remaining pulp space was backfilled with thermoplasticized gutta-percha (E and Q Plus-Meta Biomed Co., Ltd.) and AH Plus sealer (Dentsply; Detrey, Konstanz) by using warm vertical condensation technique. The tooth was restored with a composite resin (Z250; 3M ESPE, St. Paul, MN) and the patient was informed regarding the possible need for surgical intervention. On the 1-month recall visit, the patient reported no episodes of discomfort and/or swelling (Figure 1(c)). Periapical radiographs taken at 3- and 6-month recall visits to evaluate the periapical condition showed slight resorption of the MTA which was extruded into periapical area (Figures 1(d) and 1(e)). Clinical examination revealed the absence of any signs and symptoms, with adequate function.

### 2.2. Case 2

**Figure 2:**
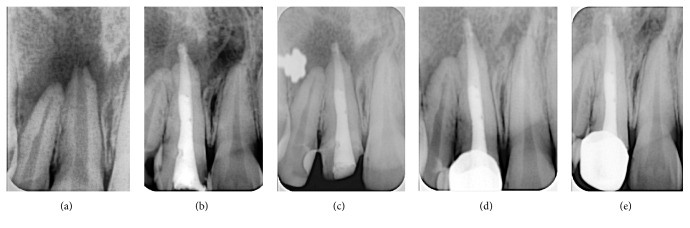
(a) Preoperative IOPA, (b) unintentional extrusion of MTA from apical foramen, (c) 1-month follow-up, (d) 3-month follow-up, and (e) 6-month follow-up.

A 30-year-old female patient reported to the Department of Conservative Dentistry and Endodontics with the complaint of pain associated with the left maxillary central incisor (tooth #21). She presented with a history of trauma to tooth #21, about 18–20 years ago. Tenderness on palpation was noted in the vestibule area. Radiographic examination revealed open apex of tooth #21 associated with a large periapical lesion of about 8 mm in diameter (Figure 2(a)). The tooth #22 was found to be vital on electric pulp testing and cold test. A diagnosis of chronic apical abscess with incompletely formed apex of tooth #21 was made, and nonsurgical endodontic treatment was planned. Access cavity and cleaning and shaping procedures were performed as described earlier. A calcium hydroxide paste (RC Cal; Prime Dental Products Pvt. Ltd., Thane, India) was placed using a size #40 Lentulo spiral (Maillefer, Ballaigues, Switzerland) to the working length and rotated at 500 rpm using a handpiece mounted on a speed and torque control machine (X-smart, Dentsply, Tulsa Dental, Tulsa, OK, USA), as an intracanal medicament for a week.

One week later, the canal of tooth #21 was irrigated alternately with 5.25% NaOCl (Prime Dental Products Pvt. Ltd., Thane, India) and 17% EDTA (Prime Dental Products Pvt. Ltd., Thane, India) and dried with sterile paper points. A one-step MTA (ProRoot MTA; Dentsply Tulsa Dental Specialities, Johnson City, TN, USA) apexification was performed as described in Case 1. Despite adequate precautions ensured during MTA placement, there was some extrusion of MTA (Figure 2(b)) into the periapical tissues. The patient was kept under observation for any signs or symptoms of clinical discomfort. After 15 days, the patient was recalled and assessed. No history of discomfort was reported. The root canal was then backfilled with thermoplasticized gutta-percha (E and Q Plus-Meta Biomed Co., Ltd.) and the access cavity was restored with a composite resin. The patient was scheduled for follow-up at 1- and 3-month intervals (Figures 2(c) and 2(d)). The patient was seen to be asymptomatic. Thereafter, at 6-month recall, a decrease in size of the periapical radiolucency was noted on periapical radiograph (Figure 2(e)).

### 2.3. Case 3

**Figure 3:**
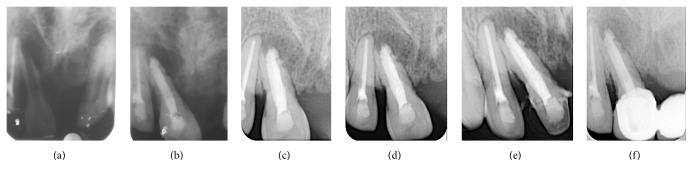
(a) Preoperative IOPA, (b) unintentional extrusion of MTA from apical foramen, (c) 1-month follow-up, (d) 2-month follow-up, (e) 3-month follow-up, and (f) 6-month follow-up.

A 17-year-old male patient reported to the Department of Conservative Dentistry and Endodontics with aesthetic concerns resulting from the discolouration of the maxillary right central incisor. The medical history was noncontributory. The patient's history included a traumatic injury in that region 5-6 years back. Extraoral examination revealed normal soft tissue structures with no apparent pathosis. On intraoral examination, a sinus tract was located in association with the maxillary right anterior region. Evaluation of periapical radiographs showed a large radiolucent lesion with well-defined margins around the root of tooth #11 along with an open apex and root resorption (Figure 3(a)). The tooth was tender to percussion. Electronic pulp testing (Electric Pulp Tester; Parkell, Farmingdale, NY) and cold application with a carbon dioxide snow (Odontotest; Moyco Union Broach, York, PA) were negative. On the basis of these findings, the patient was diagnosed as having a large periradicular lesion in the left maxillary incisor with a necrosed pulp and an open apex.

At the first visit, root canal treatment was initiated on tooth #11 under rubber dam isolation, and the access cavity was prepared. Necrotic pulp tissue was extirpated, and the working length was estimated as being 1 mm short of the radiographic apex. The drainage of pus was noted. The cleaning shaping procedures were performed as described earlier. A calcium hydroxide paste (RC Cal; Prime Dental Products Pvt. Ltd., Thane, India) was placed using a size #40 Lentulo spiral (Maillefer, Ballaigues, Switzerland) to the working length and then rotated at 500 rpm using a handpiece mounted on a speed and torque control machine (X-Smart, Dentsply, Tulsa Dental, Tulsa, OK, USA), as an intracanal medicament for one week. Sterile cotton pellets were placed into the access cavity before sealing it with temporary filling material (Cavit, 3M/ESPE, Seefeld, Germany). When the patient reported back after 1 week, the tooth was seen to be asymptomatic. The tooth was reopened, and calcium hydroxide paste was removed. The canals were irrigated alternately with 5.25% NaOCl (Prime Dental Products Pvt. Ltd., Thane, India) and 17% EDTA (Prime Dental Products Pvt. Ltd., Thane, India) with a final rinse with normal saline solution and dried with sterile paper points. MTA apexification was performed as described earlier, but unintentionally a considerable amount of MTA was seen to have extruded into the periapical region, when checked on periapical radiograph (Figure 3(b)). A moistened cotton pellet was placed in the root canal and the patient was kept under observation for 15 days. The access cavity was sealed with temporary filling material (Cavit, 3M/ESPE, Seefeld, Germany). At the subsequent appointment, the cotton pellet was removed, and after verifying the clinical signs and symptoms and the set of the MTA, the remaining pulp space was obturated with gutta-percha (Dentsply Maillefer, Dentsply India Pvt. Ltd., Haryana) and AH Plus sealer (Dentsply; Detrey, Konstanz) by using the lateral condensation technique. The tooth was restored with a composite resin.

At the one-month follow-up appointment (Figure 3(c)), the tooth was asymptomatic. The 3- and 6-month follow-up periapical radiographs showed gradual healing of the periapical lesion along with resorption of the MTA (Figures 3(e) and 3(f)).

## 3. Discussion

The low cytotoxic potential of MTA explains its wide range of applications in endodontics [4]. It has been shown that the human osteoblasts were able to attach and proliferate on MTA surfaces, suggesting the suitable use of this material adjacent to bone [11]. In addition, MTA is responsible for induction of hydroxyapatite crystal on its surface on contact with tissue fluids, which makes it a bioactive material [12]. The material derives its biocompatibility, potential for hard-tissue induction and sealing ability from this phenomenon. Also, these characteristics make MTA a suitable material to be used for apexification.

For the present case series, undesirably so, during apexification procedure, an appreciable amount of MTA was extruded into the apical lesion. It may be contemplated that the wide apical foramen might have resulted in the dislodgement of MTA through it, or it might have been pushed actively beyond apical foramen as a result of condensation pressure during placement. The healing outcomes of these three cases were very similar and favorable as compared to the materials that contain paraformaldehyde, calcium hydroxide, and/or eugenol. The outcomes of these present cases mirrored the results from animal studies as well [13].

In this case series, calcium hydroxide was used as an intracanal medicament before MTA placement to allow adequate disinfection of the root canal without the risk of compromised root strength. Researchers have proved that calcium hydroxide pastes do not significantly affect the sealability of MTA [14]. In contrast, other authors have suggested that the remnants of calcium hydroxide on the canal walls react to form calcium carbonate that can interfere with the seal [15]. The acidic pH has a negative influence on the setting characteristics of MTA [4]. In fact, the combination of MTA and calcium hydroxide in apexification procedures has been shown to favorably influence the regeneration of the periodontium [14].

The studies on the effectiveness of intracanal placement techniques of calcium hydroxide state that the most effective delivery of calcium hydroxide was achieved when the paste carriers were introduced to working length [16]. Therefore, in all the three cases described here, Lentulo spiral was used to deliver the intracanal medicament to the working length. The use of a Lentulo spiral in cases of large periapical lesions especially associated with teeth having wide apical diameters carries a risk of medicament extrusion. To avoid this, the rotation speed during the spiral filling technique was limited to 500 rpm. Hence, in none of the cases described here, calcium hydroxide was seen to be extruded, which was confirmed radiographically.

On the other hand, the use of calcium hydroxide as an intracanal medicament prior to MTA apexification has been related to the extrusion of the MTA material [15]. Therefore, use of calcium hydroxide might have resulted in the apical extrusion of the MTA material. Witherspoon and Ham [17] have reported successful and clinically effective one-visit MTA apexification procedures when MTA apical plug was placed with an orthograde approach, irrespective of the application of any internal matrix or intracanal medicament.

Although MTA is a nontoxic material, it should be restricted to the root canal space, and the condensation pressure is required to be considerably reduced in order to prevent MTA from being pushed beyond the apex [18]. A resorbable matrix has been suggested for the easy length control and for prevention of overfilling [19]. Calcium sulfate has been used as an internal matrix. However, the use of CollaPlug (Zimmer Dental, Warsaw, In, USA) as an apical matrix did not significantly prevent extrusion or improve the sealing ability of MTA [20].

It was demonstrated in this case series that there were no undue complications when MTA was applied as a root canal filling and extruded into the periradicular lesion associated with necrotic pulps. The teeth were continuously asymptomatic for the entire duration after the MTA filling was extruded. There was no complete resorption of the filling material, although gradual periradicular healing and slight resorption of extruded material were observed. Osseous repair was also noted. Indeed, the absence of clinical and radiographic symptoms justified the initial nonsurgical approach.

## 4. Conclusion

The clinical findings of the present cases imply evidence that MTA favors the apexification and periapical healing, even though a considerable amount of this material had unintentionally been extruded. The follow-up observations at 3- and 6-month intervals support the fact that the extruded material does not act as a hindrance for the healing of periapical tissues. The present case series also reinforces the importance of cautious placement of MTA apical plugs and does not recommend intentional overfilling of MTA into the periapical lesion in any clinical scenario. Revascularization procedures can be considered for such cases to obtain biological healing of the periradicular tissues, which would thereby eliminate the likelihood of material extrusion.
